# Pathology of the Laryngeal Vagus

**Published:** 1887-08

**Authors:** 


### PROF. BROWN-SEQUARD ON THE PATHOLOGY OF THE LARYNGEAL VAGUS.

WHATEVER of scientific truth may eventually be established concerning the reflex relationship of the cervical region with the brain and bulb, now set forth by Prof. Brown-Sequard, there can be no question of the remarkable suggestiveness of his conclusions. He showed some years ago that puncture of a certain region of the floor of the fourth ventricle, in close juxtaposition to the vaso-motor and cardiac centers, but not identical with them, has the effect of reducing to the lowest possible ebb the nutritional changes of the organism, so that the blood in the veins runs as red as that in the arteries; for the protoplasmic tissues neither absorb oxygen nor disengage carbonic acid, being, in fact, in a state of suspended animation. But this is not the only cessation of activity that may be brought about as the effect of puncture of the floor of the fourth ventricle. The activity of the cerebral cortex may also be suspended by a similar operation; and actual death may also occur. To sum up in a few words the latest conclusions of the distinguished experimental pathologist, we may state that the skin of the neck covering the larynx has, like stimulation of the larynx, though in a less degree, the power of inhibiting the sensibility of the body, and, further, that mechanical irritation of the larynx, trachea, and perhaps of their subjacent skin, possesses the power of causing death, in the same way as though the bulb and medulla oblongata were irritated. These are extremely important conclusions, and it will be well to examine the evidence on which they are founded. The following is the gist of Prof. Brown-Sequard's communication to the Académie des Sciences.

It has often excited surprise that suicide by cutting the throat should be carried out with such determination, and apparently at the cost of enormous suffering, indicating an almost superhuman courage. The surprise, however, would be considerably lessened, if it were true, as is now asserted, that mechanical irritation of the skin covering the larynx, as well as the larynx itself, causes total analgesia, as seems possible from the remarkable experiments Professor Brown-Sequard made some four years ago. After having made a longitudinal incision through the skin of the middle line or transversely from one side to the other, in the anterior cervical region, the Professor observed in a great number of experiments, especially in dogs and monkeys, that he could lay bare, cut, bruise, galvanize and even burn the various structures in the anterior two-thirds of the neck without causing any great pain, and sometimes without appearing to cause any pain whatever. Such facts —verified hundreds of times during the last five or six years—prove that these structures have the inhibitory property of causing general analgesia, though in a varying degree, according to the precise structure stimulated—viz.: (1) The maximum effect is produced by stimulation of the mucous membrane of the larynx—*i. e.*, of the parts supplied by the terminal ramifications of the superior laryngeal nerves. (2) In a less degree irritation of the trunks of these nerves, and in a still less degree of the trunks of the vagi above the point of emission of the superior laryngeal nerves, causes the same result to appear. (3) A transient analgesia of variable completeness may be caused by ligature of the trachea. (4) The minimum effect is caused by the irritation of the skin covering the throat, and especially of that over the larynx proper. Although incision of the various structures is the most effective in bringing about the analgesic effects, it is not the only irritation that possesses this property. The trigeminal and other sensory cranial or spinal nerves in their trunks or ramifications do not appear to be endowed with the special power that the vagus nerves and the nerves of the cervical region possess. Surgeons performing tracheotomy during the asphyxia of a patient may be incorrect in supposing that the asphyxia confers immunity from pain, for doubtless the analgesia is partly owing to the very incision of the skin of the front of the neck. Other experiments on animals seem to show that stimulation of the anterior cervical region, especially the larynx, but also the trachea, and probably the superjacent skin, has the power of stopping the heart, inhibiting the respiration, and suspending consciousness. When Prof. Brown-Sequard has killed dogs by cutting their throats, he has found that nearly always, if not always, death occurred without convulsions, without agony, in a state of perfect syncope, permitting the protoplasmic tissues of the body to preserve for a long time their special properties: the blood running red from the arteries to the veins, and thus presenting an absolute contrast to death due to asphyxia, where the arteries are filled with black blood.—*The London Lancet.*

